# Decision making from economic and signal detection perspectives: development of an integrated framework

**DOI:** 10.3389/fpsyg.2015.00952

**Published:** 2015-07-08

**Authors:** Spencer K. Lynn, Jolie B. Wormwood, Lisa F. Barrett, Karen S. Quigley

**Affiliations:** ^1^Interdisciplinary Affective Science Laboratory, Department of Psychology, Northeastern UniversityBoston, MA, USA; ^2^Department of Psychiatry, Martinos Center for Biomedical Imaging, Massachusetts General HospitalBoston, MA, USA; ^3^Edith Nourse Rogers Memorial Veterans HospitalBedford, MA, USA

**Keywords:** signal detection theory, prospect theory, judgment and decision making, risk, uncertainty

## Abstract

Behavior is comprised of decisions made from moment to moment (i.e., to respond one way or another). Often, the decision maker cannot be certain of the value to be accrued from the decision (i.e., the outcome value). Decisions made under outcome value uncertainty form the basis of the economic framework of decision making. Behavior is also based on perception—perception of the external physical world and of the internal bodily milieu, which both provide cues that guide decision making. These perceptual signals are also often uncertain: another person's scowling facial expression may indicate threat or intense concentration, alternatives that require different responses from the perceiver. Decisions made under perceptual uncertainty form the basis of the signals framework of decision making. Traditional behavioral economic approaches to decision making focus on the uncertainty that comes from variability in possible outcome values, and typically ignore the influence of perceptual uncertainty. Conversely, traditional signal detection approaches to decision making focus on the uncertainty that arises from variability in perceptual signals and typically ignore the influence of outcome value uncertainty. Here, we compare and contrast the economic and signals frameworks that guide research in decision making, with the aim of promoting their integration. We show that an integrated framework can expand our ability to understand a wider variety of decision-making behaviors, in particular the complexly determined real-world decisions we all make every day.

## Introduction

Decision making is studied from multiple perspectives, including computational modeling, economics, epidemiology, neurobiology, and psychology. Across these disciplines, decision making is studied predominantly from two frameworks. The economic framework, formalized by utility theory (e.g., Friedman and Savage, 1948) and prospect theory (Kahneman and Tversky, 1979; Tversky and Kahneman, 1992), emphasizes how people respond to variability in the value of a decision outcome, called economic risk. The signals framework, formalized by signal detection theory (SDT, Green and Swets, 1966), emphasizes how people respond to variability in what something “looks like,” called perceptual uncertainty or sometimes signal-borne risk (Lynn et al., 2005; Lynn, 2010). In its most basic sense, the commonality across the two frameworks is the selection of a choice from among options. Nonetheless, the economic and signals frameworks have remained largely isolated from one another, likely the result of these frameworks' orthogonal foci on economic vs. signal-borne risk, respectively. Here, we discuss these two decision-making frameworks with the aim of comparing and contrasting their elements. Our goal is to provide an integrated framework for decision making that unites the two existing frameworks, thereby promoting the identification of shared questions of interest, shared methodologies, relevant results across disparate disciplines, and identification of new avenues for research.

The integration of these frameworks is also critical because many important real-world decisions involve both economic and signal-borne risk, and yet research aimed at understanding such decisions has focused almost exclusively on one type of risk in isolation of the other. Cancer detection, for example, is a high-stakes decision in which the influence of signal-borne risk has received a great deal of attention. For example, Swets and colleagues have described as a signal detection issue the process of judging whether possible tumors imaged by mammography are malignant (e.g., Swets, 1998; Swets et al., 2000). Cancer has also been viewed within the economic framework where, for example, studies have examined how patients estimate and rank their preferences among treatment options (e.g., Saigal, 2009; for a meta-analysis of subjective utility of treatment option in prostate cancer, see Bremner et al., 2007). However, there is little attention to economic risk in recognized signal detection issues, such as cancer detection, or of signal-borne risk in recognized economic issues, such as choosing among treatment options for a particular cancer. Nonetheless, both types of risk are present. For example, clinicians make biopsy recommendations based on ambiguous signs of pathology (perceptual uncertainty or signal-borne risk) and the outcome of a given biopsy may be more unpredictable for one patient than for another (outcome variability, or economic risk). Thus, focus on one source of risk to the exclusion of the other may miss important interactions that influence real-world decisions.

Interactions between signal-borne and economic risk are also likely important for understanding the more frequent decisions that people make in their lives every day. Consider a decision that most people make multiple times a day–choosing what to eat. In Western countries where food is generally plentiful, food decisions have complex subjective outcome valuations. We presumably want food that tastes good and is also healthy. These values can be at odds with one another, and we sometimes feel a need to choose food that is less tasty, but more healthy. Additionally, perceptual uncertainty plays a role in food choice. Determining whether food is healthy can be a difficult perceptual task. For example, Americans now frequently consume prepared foods because of their greater convenience. The US government requires labeling of most prepared foods with a list of ingredients and their protein, fat, and carbohydrate content. Nonetheless, it can be hard to discern the proportion of nutritious content (e.g., protein, dietary fiber) relative to less nutritious content (e.g., fat, sugar), even for the careful consumer. This perceptual uncertainty is higher still for foods without content labels such as at restaurants, where pictures or other food item labels on a menu do not indicate the healthfulness of the foods. Perceptual uncertainty also plays a role in determining whether food is or is not spoiled, a different kind of “healthful” food. We cannot always distinguish spoiled food by eye or by smell, as illustrated by outbreaks of salmonella in putatively “healthy” vegetables (e.g., spinach, lettuce, tomatoes, and basil; CDC, 2010). An integrated framework would be well-suited to framing research questions about these kinds of decisions and the subjective perceptual and economic evaluations that they entail.

In this article, we propose just such an integrated framework, the Integrated Signals and Economic (ISE) Framework, which provides a mathematical model for studying decisions that involve both economic and signal-borne risk within a single decision-making framework. However, we begin by providing background information on the two predominant, existing frameworks of decision making—the economic and signals frameworks—for readers who may be unfamiliar with one or both (see Existing Decision-making Frameworks). In these background sections, we provide a specific real-world decision-making example that enables us to contrast features emphasized by each of the frameworks and thus to point out the strengths and limitations of each. Next, in Integrating the Signals and Economic Frameworks, we integrate the two existing frameworks into a single, new overarching framework, including a mathematical description. In the final section (Research Agenda Suggested by the ISE Framework), we provide additional examples of research questions that could be better addressed using the new integrated framework, leveraging insights from this integrated framework to address more complex decisions like those often made in the real world.

## Existing decision-making frameworks

### Terminology

Before providing more detailed discussion of the economic and signals frameworks of decision-making, we first define terminology used by each framework, with an emphasis on comparing and contrasting their differing terminology, and in some cases, how they use identical terminology to mean something different (Table 1). Showing how these terms differ both contrasts the economic and signals frameworks and provides an important step toward their integration by allowing better communication and understanding among researchers from different theoretical backgrounds.

**Table 1:** **Comparison of terms used in the economic and signals frameworks**.

**Term**	**Economic framework**	**Signals framework**
Risk	Variability in costs or benefits. In some uses, that variability must be known to the decision maker^a^	Likelihood of accruing a cost
Uncertainty	Outcomes that occur with a probability less than 1. In some uses, variability in a payoff that is unknown to the decision maker^a^	The same signal value can be an exemplar of both the target and foil categories
Outcome	The event that follows making a decision (e.g., the number rolled on a die), or the event's payoff	A correct detection, missed detection, false alarm, or correct rejection event; this is independent from its associated payoff
Value	The payoff (benefit or cost) accrued from a decision	The physical measurement of a signal, or the payoff (benefit or cost) accrued from a decision
Base rate	The probability associated with a specific outcome or payoff	The relative encounter rate with the target vs. foil categories

a *In the economic framework, uncertainty is often used to mean that an outcome value occurs with some probability less than 1 (i.e., it is not necessarily certain to happen). However, uncertainty has also been used to refer to ambiguity of the probabilities associated with an option's various possible outcome values (i.e., uncertainty about the probabilities themselves; Knight, 1921; Chua Chow and Sarin, 2002; Volz and Gigerenzer, 2012; see also Bland and Schaefer, 2012). Thus, some researchers utilize a stricter meaning for uncertainty, meaning unknown (to the decision maker) outcome value variability. If the variability is known by the decision maker the outcome is risky; if it is unknown the outcome is uncertain. Here, we do not distinguish known from unknown variability. Because it separately parameterizes all payoff and probability elements, the ISE Framework offers the potential to flexibly model the effect of known vs. unknown variability on decision making*.

### The economic framework

In the economic framework (e.g., Kahneman et al., 1982), a decision maker must choose one of two (or more) options, commonly referred to as “prospects,” “gambles,” or “wagers.” The options typically differ in the variability of their potential outcome values, or payoffs. The options may have monetary outcome values (e.g., money gained by choosing one option or another), although the economic framework is not limited to decisions about money. For example, the outcome values could be lives saved by choosing to administer a vaccine or not. Consider a typical monetary decision-making example: A decision maker is asked to choose between $50 for certain (Option A) or a 50% chance of getting either $25 or $75 (Option B). In this example, Option A has no outcome value variability; selecting Option A will always result in the same payoff. Option B, however, has outcome value variability, although on average, it has the same $50 expected payoff as Option A. Thus, the economic framework focuses on variability in the costs and benefits accrued as a result of a decision. Within the economic framework, decisions typically do not involve perceptual uncertainty about the options–for example different options are perceptually distinct and clearly defined. Major emphases of research within the economic framework include the effects of outcome value variation on choice (e.g., Verplanken and Holland, 2002; Mcclure et al., 2004), the effect of context or framing on sensitivity to outcome value variation (Kahneman and Tversky, 1979; Kühberger, 1998; Windmann et al., 2006), and the neuroanatomical and functional correlates of economic decision making (e.g., Knutson et al., 2005; Kable and Glimcher, 2007).

#### An example

Imagine that you have the option to ride a bicycle to work or drive a car. Let us assume that you prefer to drive if it will be a rainy day, but you prefer to bike if it will be a sunny day and that getting caught on your bicycle in heavy rain is worse than getting caught in light rain. Where you live, there is a 50% chance that it will rain on any given day. On half of the days when it rains, it is a light rain. On half of the days when it rains, it is a heavy rain. Every work day, you make a decision, choosing either to commute by car or by bicycle.

This example has features typical of a decision posed within the economic framework. Namely, there are two options to choose from, “Bike” or “Drive.” Each option results in an outcome that occurs with some probability, and has an associated value that will accrue. Let us represent the value of the options as points, as a proxy for something like “enjoyment,” and say that if you choose to bike, there is a 50% chance of a 100 point gain (it is a sunny day), a 25% chance of a 10 point loss (a light rain) and a 25% chance of a 90 point loss (a heavy rain). If you choose to drive, there is a 50% chance of a 100 point gain (it is a rainy day) and a 50% chance of a 50 point loss (it is a sunny day).

According to expected value theory, a widely used formalism within the economic framework (Friedman and Savage, 1948; Keeney and Raiffa, 1976), the value a decision maker can expect to accrue for choosing a particular option, *EV*(Option), is given by the sum of the values (*v_i_*) of each of its possible outcomes (for *i* = 1 to *n*) weighted by the probability (*p_i_*) with which that outcome will occur:

(1) $$EV(\text{Option})=p_{1}v_{1}+p_{2}v_{2}+\ldots +p_{n}v_{n}$$

An option, then, results in any one of several possible outcomes of different value and probability. Some outcomes will have positive values (benefits) and some have negative values (costs). The *expected value* of an option is the average value accrued over a series of choices of that option. The probabilities of outcomes 1 to *n* must sum to 1. In expected value theory, a decision maker should choose the option with the highest expected value. If the expected values among options are equal (as they are here), then a preference for risk proneness or risk aversion can dictate choice (biking or driving, respectively).

**Figure 1:**
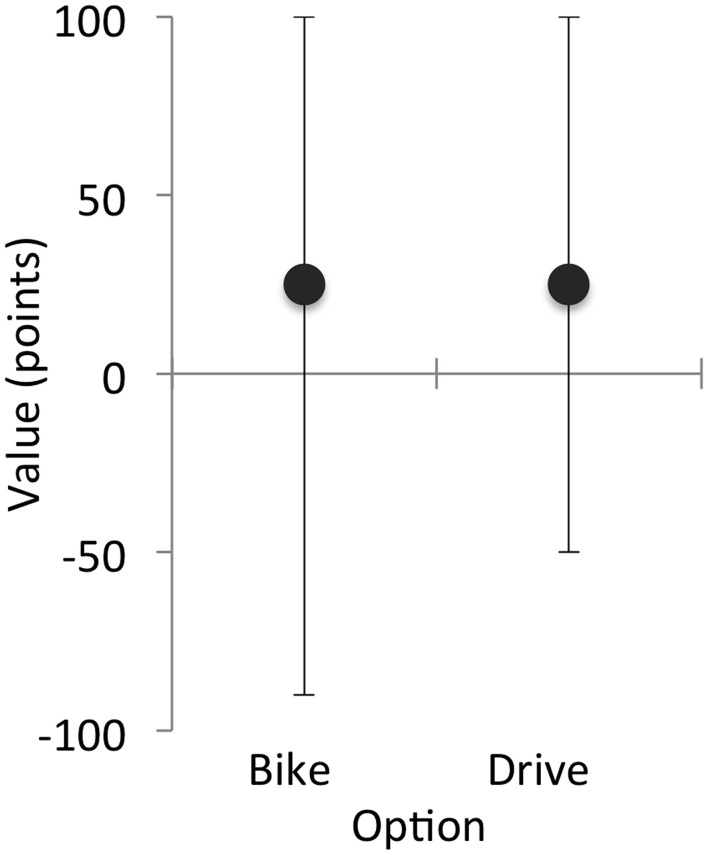
**Expected value (circles) and minimum and maximum possible payoffs (error bars) for a hypothetical choice to bike or drive to work**.

In the current example, the expected value of the options is equal, but one option has greater variability in its outcome values than the other. *EV*(bike) = (0.5 × 100 points) + (0.25 × −10 points) + (0.25 × −90 points) = 25 points. *EV*(drive) = (0.5 × −100 points) + (0.5 × 0.5 × 100 points) + (0.5 ×0.5 × 100 points) = 25 points. In this example, both options involve economic risk: the value accrued by choosing either option could be beneficial or costly, depending on the weather. However, the costs of biking might be high or low, depending on how hard it rains. Thus, the option to bike has additional risk of potential losses that the drive option does not have (Figure 1).

#### Psychological influences on parameter estimates

Within the economic framework, utility theory and prospect theory represent modifications of expected value theory that incorporate psychological influences on decision making. Utility theory (e.g., Friedman and Savage, 1948) arose as a revision of expected value theory when considerable empirical evidence accrued that failed to fit the predictions of expected value theory. In particular, researchers observed that objective outcome values do not have a strongly linear relationship with subjective outcome values–a given amount of money is not worth the same thing to different people or to the same person in different contexts. This non-linearity resulted in poor prediction of choice behavior using expected value theory. In utility theory, objective outcome values (typically monetary values) are transformed into subjective values (utilities) according to a utility function, *u(v_i_)*. The expected value of an option in utility theory is given by:

(2) $$\hat{U}\left(\text{Option}\right)=p_{1}u\left(v_{1}\right)+p_{2}u\left(v_{2}\right)+\ldots +p_{n}u\left(v_{n}\right)$$

**Figure 2:**
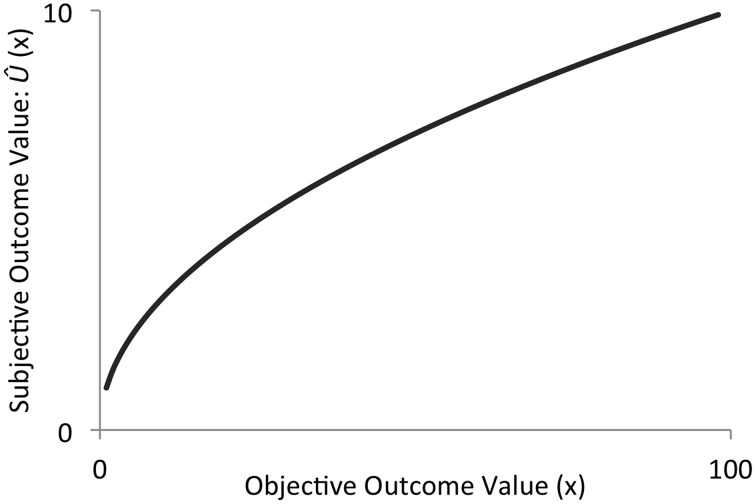
**A hypothetical utility function**. Utility changes as a convex function of absolute objective value.

The typical empirically-derived shape of *u*(*v_i_*) captures the fact that subjective value changes rapidly at low objective values and does not change as rapidly as objective value increases (Figure 2).

**Figure 3:**
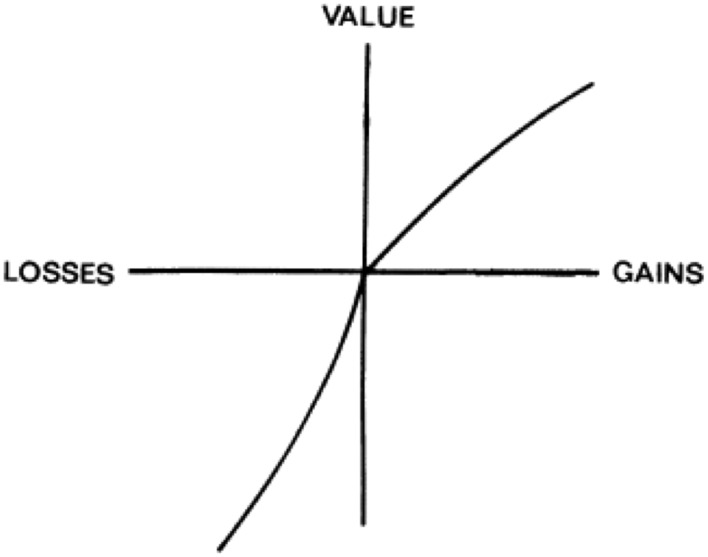
**A hypothetical value function from prospect theory**. The value function is generally convex for gains and concave for losses, and steeper for losses than for gains. From Kahneman and Tversky (1979), with permission from the Econometric Society.

Despite the advancements of utility theory over expected value theory in dealing with psychological influences on decision making, researchers continued to observe human behavior that failed to adhere even to the predictions of utility theory. To address these shortcomings, prospect theory (Kahneman and Tversky, 1979; Tversky and Kahneman, 1992) improved upon estimates of subjective value and addressed people's seemingly irrational use of base rates (the relative probabilities of alternative outcomes in a decision). Prospect theory shares features with utility theory. Both utilize a subjective value function, but in prospect theory the shape of this function is altered, and termed a “value function” instead of a “utility function.” Unlike a utility function, the value function, *V(v_i_)* is defined not by absolute subjective values, but by deviations from a specific reference point (typically one's current assets). The value function is generally convex for gains and concave for losses, and steeper for losses than for gains (Figure 3). In addition, prospect theory proposes that probabilities (typically given explicitly for each option) are transformed into subjective decision weights (analogous to how objective outcome values are transformed into subjective outcome values) using a probability-weighting function, *d(p_i_)*. The probability-weighting function is meant to account for individuals' so-called “irrational” treatment of extreme probabilities. For example, the probability-weighting function defines decision weights that are greater than the stated objective probabilities when those stated probabilities are very low. Likewise, the function defines decision weights less than the stated probabilities when those probabilities are very high. The expected value of an option in prospect theory is:

(3) $$\begin{array}{l} P\left(\text{Option}\right)=V\text{​}\left(v_{1}\right)d\left(p_{1}\right)+V\text{​}\left(v_{2}\right)d\left(p_{2}\right)+\ldots \\ \;+V\text{​}\left(v_{n}\right)d\left(p_{n}\right) \end{array}$$

The types of decisions posed within the economic framework can be quite similar to problems faced by decision makers outside the laboratory, e.g., maximizing returns on investments, tradeoffs made when buying different car models. Nonetheless, experimental tests of how these decisions are made typically do not implement perceptual uncertainty, which is the major focus of the signals framework. For example, investment decisions posed in written vignettes delineate explicit and distinctive options, which may have more or less variable payoffs (e.g., in terms of money earned, lives saved). Similarly, in a card-playing task, visibly distinct card decks ensure that a decision maker will draw a card from one or the other of the decks displayed. Although the decision maker may be uncertain about the value of the card to be drawn, there is no uncertainty about whether the card is from one deck vs. the other. Researchers solely utilizing the economic framework to understand decision making will neglect how perceptual uncertainty can affect decision processes.

### The signals framework

**Figure 4:**
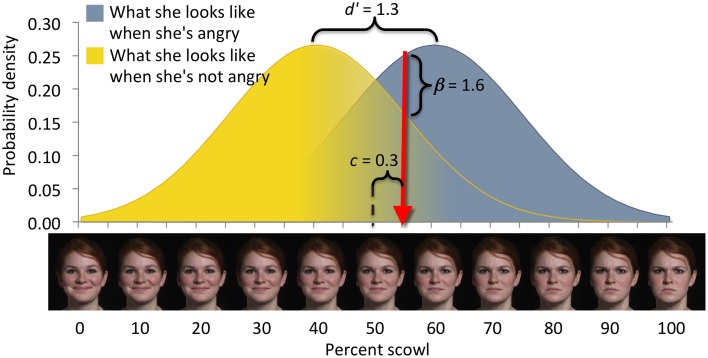
**Elements of the signals framework (after Lynn and Barrett, 2014)**. In emotion perception, for example, facial expressions are evaluated by one person (the perceiver) to determine the emotional state of another person (the sender). Signals, depicted on the *x*-axis, comprise two categories: targets (defining, e.g., what the sender looks like when she is angry) and foils (defining, e.g., what the sender looks like when she is not angry). Signals from either category vary over a perceptual domain such as “scowl intensity.” Any signal (i.e., a particular scowl intensity) can arise from either category, with a likelihood given by the target and foil distributions. Perceivers therefore, experience uncertainty about the category membership of any particular signal. Here, the perceiver responds to facial expressions to the right of criterion (red arrow) as if they were angry, and to facial expressions to the left of criterion as if they were not angry. Perceivers make a decision between two options and the perceptual uncertainty yields four possible outcomes: (1) Classifying a stimulus as a target when it is a target is a correct detection. (2) Classifying a stimulus as a target when it is a foil is a false alarm. (3) Classifying a stimulus as a foil when it is a target is a missed detection. (4) Classifying a stimulus as a foil when it is a foil is a correct rejection. Measures of sensitivity (e.g., *d'*) characterize perceptual uncertainty, depicted here as overlap of the target and foil distributions. Measures of bias (e.g., *c* or *beta*) characterize the decision criterion's location in the perceptual domain. Sensitivity and bias are derived from the numbers of correct detections and false alarms committed over a series of decisions (Macmillan and Creelman, 2005). Perceptual uncertainty causes the perceiver to make mistakes regardless of his or her degree of bias; missed detections cannot be reduced without increasing false alarms.

In the signals framework (Green and Swets, 1966; Lynn and Barrett, 2014), a perceiver classifies a percept as one of two possible options. In a detection decision, the perceiver decides if the percept was present (i.e., the “signal” option) or absent (i.e., the “noise” option). For example, a perceiver might be asked to determine whether a tone is present or not on each trial of an auditory task in which some trials contain non-tone white noise and other trials contain a tone of variable acoustic intensity embedded in white noise. In an identification decision, the perceiver decides if the percept (a “signal”) is an exemplar of one category (Option A) or another (Option B). For example, on each trial of an emotion perception task, a perceiver might be asked to determine whether an ambiguous facial expression communicates a state of happiness or anger. In both detection and identification paradigms, the two options span different but overlapping ranges of a single, continuous, perceptual dimension, such as decibels of acoustic energy or expressive facial features^1^ (Figure 4). Thus, the signals framework focuses on variability in the perceptual features of the options (e.g., how similar in appearance exemplars from Option A are to those of Option B). We will frame our discussion of the signals framework in terms of identification paradigms, and not further distinguish between detection and identification here [but see Green and Swets (1966) and Macmillan and Creelman (2005), for more details on this distinction].

Within the signals framework, decisions typically impose no variation in the payoffs accrued from the possible outcomes^2^ (e.g., in terms of money or points earned). In fact in many experiments, the outcomes of a decision (correct detections, false alarms, missed detections, and correct rejections; see Figure 1) are not typically assigned specific values, beyond simple feedback to indicate that the decision was correct or incorrect. When payoffs are made more specific (e.g., See et al., 1997; Lynn et al., 2012), they do not usually have probabilistic variation in the way that is modeled by the economic framework (e.g., the payoff of a correct detection does not vary from trial-to-trial). Major emphases of research within the signals framework include the factors that affect bias (weighting of costs, benefits, and probability estimates; e.g., See et al., 1997; Bohil and Maddox, 2001) and sensitivity (ability to discriminate the two options; e.g., Grinband et al., 2006; Aberg and Herzog, 2012), the neuroanatomical correlates of perceptual learning (e.g., Gold et al., 2004; Sasaki et al., 2010), and evolutionary consequences of decisions made under perceptual uncertainty (e.g., Lynn et al., 2005; Wiley, 2006).

#### An example

Let us return to the example first outlined in An Example in The Economic Framework concerning the decision to bike or drive to work. From the signals framework there is additional information available, beyond the expected value and economic risk, to help a perceiver decide which option to choose. That additional information is what the weather looks like^3^. Let us imagine that you assess the likely weather by looking out the window each morning. If it looks like it will rain, you decide to drive. If it looks like it will not rain, you decide to bike. At this point, our example becomes a signal detection issue, in addition to being an economic decision. You evaluate a signal, the weather. Deciding to drive on a rainy day (a correct detection), and deciding to ride on a non-rainy day (a correct rejection), both accrue benefits. Deciding to drive on a non-rainy day (a false alarm), and deciding to ride on a rainy day (a missed detection), both accrue costs.

In signal detection theory, the mathematical formalization of the signals framework, a perceiver makes subjective “estimates” of three parameters, though not necessarily consciously. The first parameter is the *similarity of the signal distributions*. Any signal comes from one of two distributions. Here, the distributions are “what the weather looks like when it will rain” (we will call this the category of *targets*), and “what the weather looks like when it will not rain” (so-called *foils*). Some days it is hard to tell–the sky looks the same regardless of whether it proves to be a rainy commute or not, creating perceptual uncertainty. The second parameter is the probability with which the perceiver encounters targets (rainy days) vs. foils (non-rainy days), called *base rate*. Here, we have stipulated that there is a 50% chance that it will rain on any given day. The third parameter is the *payoffs* associated with correct and incorrect decision outcomes. Translating the point values from above: a correct detection accrues the 100 point gain associated with driving on a rainy day, a missed detection accrues the 50 point loss associated with driving on a sunny day, a correct rejection accrues the 100 point gain associated with biking on a sunny day, and a false alarm accrues the average 50 point loss associated with biking on a rainy day. For now, we ignore the variation in false alarm cost due to light vs. heavy rain because application of the signals framework does not typically consider economic risk.

In signal detection theory, the perceiver is conceived of as placing a threshold, or *decision-criterion*, on the signal domain (what the weather looks like). The SDT expected value function (Swets et al., 1961) can be used to calculate the expected value of placing a criterion at any possible location on the signal domain, for a given set of parameter values:

(4) $$\begin{array}{l} EV\left(x_{i}\right)=\alpha h\text{p ​}\left[CD\right]+\alpha m\text{p ​}\left[MD\right]+(1-\alpha )a\text{p ​}\left[FA\right]\\ \;+(1-\alpha )j\text{p ​}\left[CR\right] \end{array}$$

Where:

*EV*(*x*_i_), expected value of a decision criterion at signal value *x*_i_;

α, alpha, the base rate or relative probability of encountering a signal from the target distribution; 1 −α equals the relative probability of encountering a signal from the foil distribution;

*h*, benefit of correct detection;

*m*, cost of missed detection;

*a*, cost of false alarm; and

*j*, benefit of correct rejection. Costs might be negative or simply less positive than benefits, as long as *h* > *m* and *j* > *a*.

p[*CD*], probability of correct detection, measured as the integral of the target distribution over *x* from criterion to infinity;

p[*MD*], probability of missed detection, equal to 1 − p[*CD*];

p[*FA*], probability of false alarm, measured as the integral of the foil distribution over *x* from criterion to infinity;

p[*CR*], probability of correct rejection, equal to 1 − p[*FA*].

**Figure 5:**
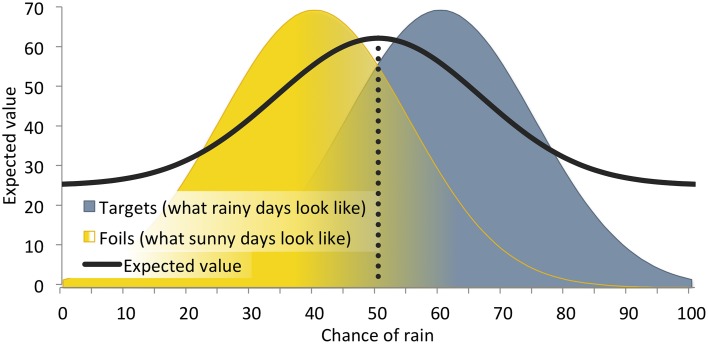
**The influence of perceptual uncertainty about the weather in a hypothetical decision about whether to bike or drive to work**. This illustration implements benefits and costs relevant to getting caught in the rain on a bicycle or not. Mean target = 60% chance of rain, mean foil = 40% chance of rain, with standard deviations = 15% chance of rain; base rate = 0.5; *h* = 100 points, *m* = −50 points, *a* = −50 points, *j* = 100 points. Given these parameter values, the optimal criterion location (drop line) indicates that a perceiver should drive to work when the chance of rain is 50% or greater. The *y*-axis for the distributions (probability density) is not shown.

The criterion location with the highest expected value is the *optimal* criterion location on average (i.e., over a series of decisions); the perceiver should drive if the weather looks worse than the optimal criterion value. Given the stipulated equivalent base rate of encountering targets vs. foils and the balanced payoff matrix in our example, the signals framework specifies a neutral bias (Figure 5). A perceiver maximizing expected value should show no tendency to select one option over the other.

## Integrating the signals and economic frameworks

If viewed solely within each of the separate decision-making frameworks, the above decision-making example (choosing to drive or bike to work; see An Example in The Economic Framework and An Example in The Signals Framework) reveals limitations that arise from these frameworks' orthogonal emphases on economic vs. signal-borne risk. Each framework highlights certain features of the decision. Using only one framework neglects decision features important to the other (see also Summerfield and Tsetsos, 2012, for additional perspective). Further, when both sources of variability are important for the decision maker, studying the features from only one of the frameworks impedes understanding of how these features interact functionally for the decision maker.

In this section, we (1) describe how perceptual uncertainty from the signals framework can be integrated into models of economic risk as an initial step toward combining these two frameworks, (2) demonstrate that consideration of economic risk in the signals framework leads to novel hypotheses and predictions for decision making, and (3) introduce a combined decision-making model, the Integrated Signals and Economic (ISE) Framework.

### Adding perceptual uncertainty to the economic framework

We can consider the emphasis on outcome value in the economic framework as modeling a special case of decisions—those in which there is no perceptual uncertainty. Imagine the following decision between two options: one option with a 50% chance of paying $35 and a 50% chance of paying $55, the other with a 100% chance of $45. To make the parallels between the economic and signals frameworks explicit, let us consider the first option the Target option and the second option the Foil option. This example can be viewed as a special case of a signal detection problem in which the target and foil options are so distinct that there is no perceptual uncertainty: the options (Target or Foil) cannot be *perceptually* mistaken for one another. If we consider these two options as equivalent to a choice between two options in the signals framework, then we recognize that the individual payoff parameters in the SDT expected value function are themselves expected value functions from the economic framework: *EV*(target) = *h* = $45 and *EV*(foil) = *j* = $45.

In the SDT expected value function, the Target and Foil options are both included in a single equation, weighted by their relative base rates of occurrence. Because, in the economic framework, the options are presented simultaneously, we take the base rates for both options to be equivalent for this example (i.e., α = 0.5). Furthermore, with no uncertainty, for the Target distribution, p[*CD*] ~ 1 and p[*MD*] ~ 0, while for the Foil distribution, p[*CR*] ~ 1 and p[*FA*] ~ 0. In signals framework terms, then:

(5) $$EV\left(x_{\text{i}}\right)=\alpha h+(1-\alpha )j$$

**Figure 6:**
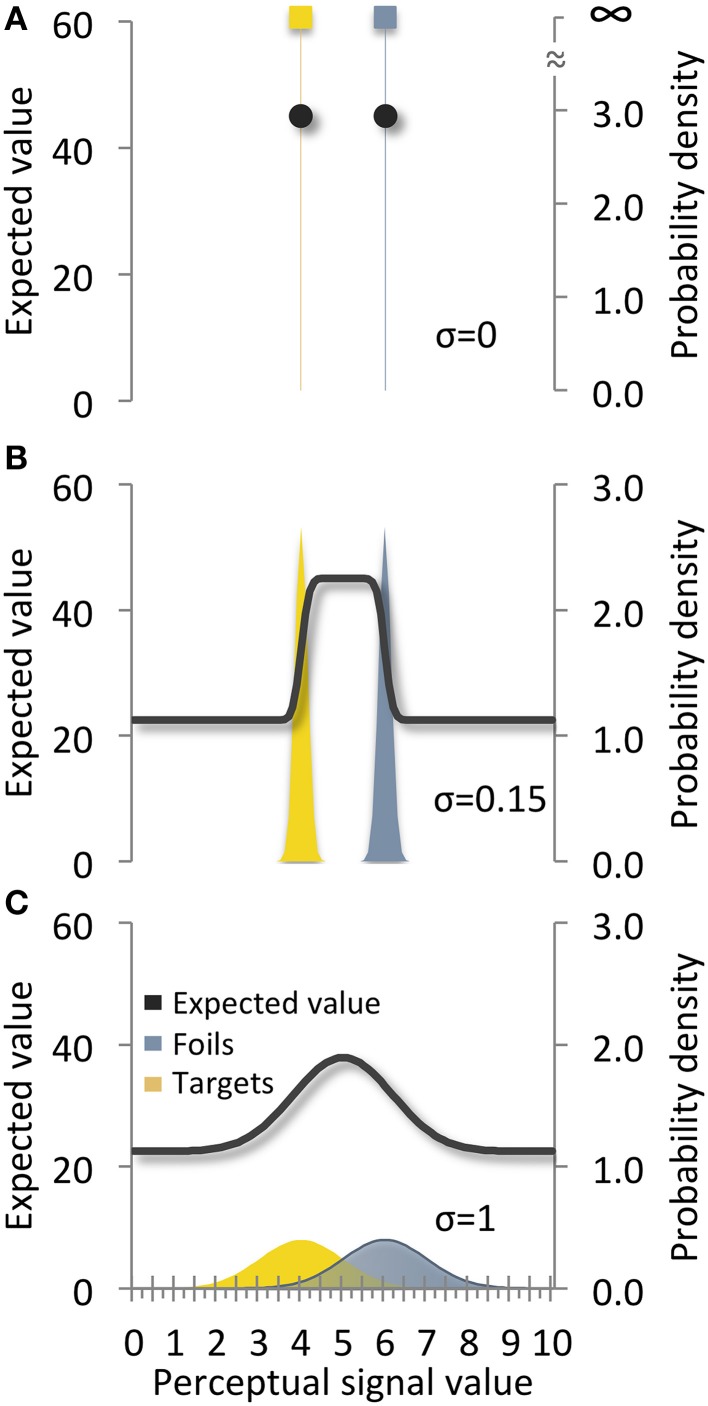
**Expected value over** ***x*** **for target and foil options (A) with no perceptual uncertainty, (B) little perceptual uncertainty, and (C) more perceptual uncertainty**. *Sigma* refers to the standard deviations of both signal distributions.

Expressed as a signal detection issue, the expected value function for a decision in the economic framework reduces to a constant over all perceptual signal values, *x*, which is the average of the expected values of the options (Figure 6A). Moreover, because there are only two perceptually distinct values of *x* (one for each option), what is generally a continuous function across all values of *x* in SDT is reduced here to two discrete points.

As perceptual uncertainty (i.e., variance in the “appearance” of the options) increases, a recognizable signal detection issue emerges (Figures 6B,C). The probability of making false alarm and missed detection mistakes becomes greater than zero and their associated costs can influence behavior, as modeled by Equation (4). When perceptual cues indicating particular options are encountered one at a time, we now have a conventional problem as posed within the signals framework.

Within the economic framework, the idea of decision *cues* is relevant to perceptual uncertainty. A cue is similar to a channel of information (e.g., Payne et al., 1992; Newell et al., 2004). For example, in Figure 4, a facial expression is a useful cue of emotional state, and a person making an emotion judgment about someone would do well to attend to that cue (i.e., to base their decisions on signals, such as scowl intensity, in that channel). However, research on cues is largely focused on cue validity and cue selection. Here, we are introducing variability of cue-values among options with the aim of modeling how that variability may influence decision making. Whereas research on cues is oriented toward how decision makers select a channel, our application of perceptual uncertainty to the economic framework is oriented toward how decision makers utilize signals within that channel.

In this section, we demonstrated that decisions studied within the economic framework can be modeled within the signals framework if one allows that many of the SDT expected value function's variables reduce to constants in the absence of perceptual uncertainty. Lacking signal distributions, these decisions reduce to a choice between the expected values of two discrete options. Despite this consideration, however, it is not accurate to claim that the signals framework captures all of the insights from both frameworks merely because it addresses a broader category of decisions. Namely, the signals framework still does not address the psychological or subjective weighting of expected value to the extent that it has been examined within the economics framework (see Psychological Influences on Parameter Estimates).

### Adding economic risk to the signals framework

While both the economic and signals frameworks have a formalization of expected value that guides decision making (Equations 1 and 4), the concept of expected value has seen additional development within the economic framework (Equations 2 and 3) that has seldom been applied within the signals framework (exceptions are Barkan et al., 1998 and Kaivanto, 2014). In this section, we model the influence of economic risk within the signals framework to highlight how their integration can lead to novel hypotheses and predictions about real-world decision making behavior.

**Figure 7:**
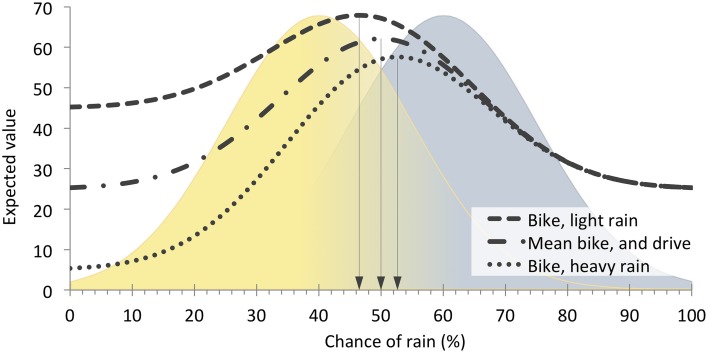
**Combined effects of perceptual uncertainty and economic variability on optimal criterion placement for the bike vs. drive example**. Variation in the false alarm cost (biking in heavy vs. light rain) generates a minimum expected value function (Bike, heavy rain) and maximum expected value function (Bike, light rain), with corresponding conservative and liberal criterion locations, relative to the average expected value (Mean bike, and drive). The mean expected value function and bell shaped regions are the same as that shown in Figure 5. Drop lines indicate optimal criterion locations for each of the three functions.

Let us return to our example of choosing whether to bike or drive to work (described in An Example in The Economic Framework and An Example in The Signals Framework). The variation in false alarm cost (i.e., the cost of mistakenly choosing to bike when it rains) represents the source of economic risk in the example, and sets the minimum (heavy rain) and maximum (light rain) expected value bounds. We can thus model variation in outcome value (i.e., economic risk) in signal detection theory as an expected-value *envelope*, bounded by minimum and maximum SDT expected value functions. Figure 7 illustrates an expected-value envelope for this example, bounded by the minimum (heavy rain) and maximum (light rain) expected value functions.

Novel predictions become apparent when the economic and signals frameworks are considered together in this way. For example, one might expect that equal variability about the mean false alarm cost would produce equal variability about the mean criterion location. Instead, however, this analysis reveals that the optimal criterion of the mean expected value function is *not* the mean of the light- and heavy-rain optimal criteria. In this example, the magnitude of the effect on behavior of a 40 point deviation from the −50-point mean false alarm cost is not the same if that 40 points reflects a relatively large loss (i.e., −90 points for a false alarm in heavy rain) as when it reflects a relative small loss (i.e., −10 points for a false alarm in light rain). This observation parallels empirical findings from the economic framework, in which “losses loom larger than gains” (Kahneman and Tversky, 1979)–the effect, on subsequent behavior, of accruing a cost is larger than the effect of accruing a benefit of equal magnitude. Our example indicates that, outside the laboratory, one contributor to such a loss bias could be perceptual uncertainty. That is, symmetric payoff variation can produce asymmetric effects on behavior in the presence of perceptual uncertainty, a phenomenon that only becomes apparent when considering interactions between economic and signal-borne risk.

In addition, Figure 7 reveals that variability in expected value is higher on the left (sunny) side of the signal domain than on the right (rainy) side for our example. We might then expect risk-averse perceivers to set a more conservative (right-sided) criterion than risk-prone perceivers because a conservative threshold would decrease exposure to variable outcome values. That is, risk-prone perceivers would be more biased toward biking while risk-averse perceivers would be more biased toward driving. Our example indicates that, while economic risk sensitivity is not typically accounted for within the signals framework, it could nonetheless influence individual differences in criterion placement. Thus, considering interactions between economic and signal-borne risk in this way may help explain findings within the signals framework where perceivers' decision criteria have failed to meet the objective parameters of the task. That is, perceivers bring their own idiosyncratic expectations to signal detection tasks, which result in observed response biases in the absence of objectively biasing payoffs or base rates (Green and Swets, 1966). Moreover, in signal detection tasks that do manipulate payoffs, humans do not easily adapt their decision criterion to the objective payoff values (Dusoir, 1975; Bohil and Maddox, 2001; Lynn et al., 2012). Individual differences in economic risk sensitivity could be one underlying explanation for the variation in criterion location seen across perceivers within a given signal detection study.

### The integrated signals and economic framework

Our position is that decisions in the context of a decision maker's daily life experiences commonly involve both signal-borne risk and economic risk. Here, we incorporate both of these types of risk into a single decision-making framework: the Integrated Signals and Economic (ISE) Framework. The ISE Framework will allow researchers to explore types of risk and uncertainty that the individual frameworks typically do not focus on. What is missing in typical approaches to decision making using the economic framework is an account of signal-borne risk. The potential outcomes associated with different options (e.g., to bike or to drive) are often cued by signals. The signals themselves are variable, and that variation can be independent of variation in the expected value (economic risk) of the decision outcome. What is missing in typical approaches to decision making using the signals framework is the idea of economic risk. Outcome values can be variable, and are themselves value functions, impacted by all the psychological factors that are the subject of contemporary research in judgment and decision making. The ISE Framework will allow for an exploration of the influence of economic risk where its role has largely been overlooked (in perceptually uncertain decisions) as well as an exploration of the influence of signal-borne risk where its role has largely been overlooked (in economically risky decisions).

A first approximation to integrating the signals and economic frameworks mathematically is to combine the subjective value function from prospect theory with the signals framework's expected value function:

(6) $$\begin{array}{l} \hat{U}\left(x_{i}\right)=\alpha V\text{​}\left(h\right)\text{p ​}\left[CD\right]+\alpha V\text{​}\left(m\right)\text{p ​}\left[MD\right]+(1-\alpha )\\ \;V\text{​}\left(a\right)\text{p ​}\left[FA\right]+(1-\alpha )V\text{​}\left(j\right)\text{p ​}\left[CR\right] \end{array}$$

Here, *V*(*h*, etc.), a prospect-theory treatment of the payoff associated with each type of decision outcome, and other variables are as defined for Equation (4).

As a second step to integrating the frameworks, we can incorporate lessons learned from the development of prospect theory about the importance of subjective weighting functions for those parameters representing probabilities associated with perceptual uncertainty:

(7) $$\begin{array}{l} \hat{U}\left(x_{i}\right)=B(\alpha )V\text{​}\left(h\right)S\left(\text{p ​}\left[CD\right]\right)+B(\alpha )V\text{​}\left(m\right)\\ \;S\left(\text{p ​}\left[MD\right]\right)+B(1-\alpha )V\text{​}\left(a\right)S\left(\text{p ​}\left[FA\right]\right)+B(1-\alpha )\\ \;V\text{​}\left(j\right)S\left(\text{p ​}\left[CR\right]\right) \end{array}$$

where:

*B*(α), a decision weight of the type applied to probability estimates in prospect theory, here applied to the relative base rate of encountering targets vs. foils.

*S*(p[*CD*], etc.), a decision weight of the type applied to probability estimates in prospect theory, here applied as an estimate of the target and foil signal distributions.

Other variables are as defined for Equation (4).

The ISE Framework generates subjective utilities of possible decision criterion locations over a continuous perceptual domain. These utilities are weighted expected values (from the SDT expected-value function). The weighting is done by functions intended to model the influence of psychological factors, such as risk sensitivity, that alter the objective values of the three environmental parameters (perceptual similarity, base rate, and payoffs; Figure 1). In this way, the ISE Framework attempts to do for SDT what prospect theory did for expected value theory and utility theory. The ISE Framework extends prior efforts to combine the two frameworks (e.g., Barkan et al., 1998; Kaivanto, 2014), which have not used the SDT expected-value function to derive criteria locations directly from underlying environmental parameters. For example, research within the economic framework documents that people over-estimate the occurrence of low probability events and under-estimate the occurrence of high probability events. Prior theoretical work modeled this effect on the SDT base rate parameter (Kaivanto, 2014). However, such probability mis-estimation presumably influences people's representation of the perceptual similarity parameter as well. The ISE Framework captures this feature.

Variability in the decision environment can be characterized at different levels of organization, e.g., the perceptual uncertainty (signal-borne risk) and outcome variability (economic risk) emphasized here, in addition to the variability in cue validity across channels described above. The functional consequences of simultaneous variability at different levels of description (e.g., for behavior, neural implementation, and cognitive impairments) are unknown. Our integration of the signals and economic frameworks can be viewed as an attempt to integrate variability of outcome values and variability of perceptual signals within a cue/channel.

An interesting question for future research is whether different sources of variability are dissociated at the neural level or whether they merely represent the same process described from distinct methodological points of view. Because the development of theoretical and computational models of decision making has proceeded largely independently within the signals and economic frameworks, so too has research examining the neural bases of decision making. Research inspired by both frameworks involves the search for a common final pathway where decision-relevant information is integrated and options are compared in some nonspecific “neural currency,” however, the two frameworks have largely focused their empirical efforts on the contributions of distinct brain regions (for a review see Summerfield and Tsetsos, 2012). Given its emphasis on distinguishing or detecting perceptual stimuli, research within the signals framework has largely focused on areas like the parietal cortex, which process dorsal stream input from primary visual areas (e.g., Treisman and Gelade, 1980; Roitman and Shadlen, 2002; Xu and Chun, 2006; Gold and Shadlen, 2007; Gottlieb and Balan, 2010). Conversely, research guided by the economic framework, with its emphasis on outcome valuation, has focused on subcortical regions involved in processing reward salience and sensitivity, largely through dopaminergic pathways (e.g., the striatum; Schultz et al., 1997; Lauwereyns et al., 2002; O'doherty et al., 2003; Nakamura and Hikosaka, 2006; Ding and Hikosaka, 2007), as well as frontal regions that receive inputs from these areas (e.g., the orbitofrontal cortex; O'doherty et al., 2001; Padoa-Schioppa and Assad, 2006; Plassmann et al., 2007; Padoa-Schioppa and Assad, 2008; Kable and Glimcher, 2009; Kennerley et al., 2009). Interestingly, however, emerging evidence suggests that regions commonly studied from the signals framework as being important for integrating sensory information are also involved in encoding information about outcome values (e.g., the parietal cortex; Platt and Glimcher, 1999; Dorris and Glimcher, 2004; Sugrue et al., 2004, 2005; Rorie et al., 2010). Similarly, current research indicates that brain regions commonly studied from the economic framework as important for value-guided decision making are also critical to making accurate perceptual category judgments (e.g., striatum; Packard et al., 1989; Eacott and Gaffan, 1991; Ashby et al., 2003). These findings highlight the need for a more general framework that can assimilate neurobiological evidence from the two distinct approaches. The ISE Framework can guide future research into the neural bases of decision making by helping researchers pose and test specific hypotheses concerning the independence of economic and signal-borne risk at the biological level.

Empirical work also will be needed to determine the shape of the new functions for the subjective weighting of base rates and signal distributions. For example, within the economic framework, changes in outcome probability are associated with more pronounced differences in decision behavior when the change in probability occurs at more extreme probabilities (e.g., changing the probability of occurrence of a payoff from 0 to 1% impacts decision behavior much more dramatically than changing the probability from 50 to 51%). Likewise, it will be important to develop and utilize subjective weighting functions for the parameters in the perceptual or signals component of the ISE Framework.

## Research agenda suggested by the ISE framework

A new research agenda for understanding decision making can emerge whereby we can model perceptual uncertainty, economic risk, and psychological influences within a single integrated framework. The new agenda promises to broaden theoretical and empirical work in decision making to more complex, and realistic decision-making circumstances.

### Modeling contextual influences on decision making

One potential scientific impact of the ISE Framework is that it permits modeling of contextual influences on decision making. Decisions occur within the larger context of a decision maker's daily life, current internal or bodily states, and past experience. The ISE Framework explicitly acknowledges that context is an important determinant of decision behavior *in vivo* that impacts perceivers' estimates of event likelihood and value (for review see Doya, 2008). Context can be broadly interpreted, and encompasses both factors external to the decision maker, such as how decision options are framed, and factors internal to the decision maker, such as the person's internal states and their behavioral tendencies or individual differences that can influence decisions in the moment. The more explicit modeling of decision-making parameters provided by the ISE Framework would expand such research by providing an approach for systematically exploring the causal mechanisms driving the influence of context on decision making.

Within the economic framework, research into the effects of context on decision making has focused on the array of individual options as a context. The collection of options among which a decision maker must choose comprises a unique context; if one option is replaced with another, the context of the decision changes. Such changes in context can lead to reversals of preference and other so-called “irrational” violations of normative assumptions when the economic risk of the options differs (Busemeyer and Townsend, 1993; Bhatia, 2013). Approaches to examining this type of context, such as decision field theory (Busemeyer and Townsend, 1993; Johnson and Busemeyer, 2005), the stochastic difference model (González-Vallejo, 2002), and the associative accumulation model (Bhatia, 2013) all model the decision maker's ability to discriminate options from one another based on differences in their economic risk (e.g., it is difficult to choose among options that have similar subjective utility). These models are context-sensitive because the discriminability among options is dependent on factors such as the individual options present (Busemeyer and Townsend, 1993; González-Vallejo, 2002) or the decision maker's learning history with the options (Busemeyer and Townsend, 1993; Weber et al., 2004).

The ISE Framework complements work in the economic framework on contextual influences by modeling how perceptual similarity can also influence context and choice. For example, Baumann and Desteno (2010) demonstrated that participants' estimates of base rates for encountering targets and foils in a threat detection task were influenced by an internal contextual variable, the participant's emotional state. Across five experiments, participants induced to feel anger were biased toward identifying neutral objects as guns, whereas participants in a more neutral emotional state were less biased. Bias was associated with the extent to which anger influenced perceivers' base rate estimates and could be eliminated by providing participants with accurate information about the number of gun trials in the task. The proposed base rate (B) weighting function of Equation (7) could be used to model the psychological effects of emotional state on objective base rate values.

Past experience is another important contextual influence on decision making that could be modeled within the ISE Framework (and see also Stewart et al., 2006, for the role of long-term memory in decisions). For example, in an experiment where radiologists performed a lung-nodule detection task, an astounding 83% missed a picture of a gorilla that was inserted into one of the images that was 48 times the size of the average lung-nodule (Drew et al., 2013). This phenomenon, known as inattentional blindness (cf. Simons and Chabris, 1999), is driven in part by individuals relying on expectations about what is likely in a given context based on their previous experiences in similar contexts. Thus, the ISE Framework could help formalize how past experience contributes to inattentional blindness by modeling past experience as weighting function B in Equation (7).

Finally, research has also demonstrated that external contexts can influence decision-making behavior. For example, the mere presence of a weapon (e.g., a gun or a knife) has been shown to make people behave more aggressively (Berkowitz and Lepage, 1967; Berkowitz, 1990, 1993). The ISE Framework can be used to examine whether the presence of a weapon influences perceptual parameters related to the decision to engage in aggressive behavior (e.g., by making other people look more aggressive or more threatening, or by increasing the estimated base rate of encountering aggressive people) or influences the subjective valuation of outcomes (e.g., by making people think behaving aggressively has a low cost or will help them avoid alternative high-cost outcomes).

### Modeling dysfunctional decision making in psychopathology

Another potential scientific impact of the ISE Framework is that it can provide a model-driven approach for characterizing and understanding dysfunctional decision making such as occurs in those with psychopathology (e.g., Redish, 2004). For example, Generalized Social Anxiety Disorder (GSAD) is characterized by exaggerated concerns about negative evaluation and rejection in social situations. People with GSAD exhibit what has been termed a “zero-miss” threat perception strategy (Quigley and Barrett, 1999) which is a pronounced tendency to respond to or recall non-threatening stimuli as threatening as a result of learning within an earlier threatening environment, and measured as a liberal bias relative to control participants. These perceptual tendencies have been described within the signals framework, but the work has largely been limited to quantifying differences in sensitivity and bias among perceivers (e.g., Gilboa-Schechtman et al., 2008). Many of the emotional and/or behavioral symptoms associated with GSAD might be understood as long-term failures to accurately estimate one or more of the parameters that describe the decision-making environment (base rate, payoffs, and perceptual similarity). In this view, it is the subjective “misestimation” of one or more parameters that results in misperceiving social stimuli as threatening, measured in the laboratory as abnormal bias and/or sensitivity (Lynn and Barrett, 2014). The ISE Framework provides a way to isolate which individually-relevant subjective weightings of decision parameter(s) are being misestimated via systematic within-subject manipulation of perceptual and decision parameters.

## Conclusions

A large class of ecologically valid, and quite common decisions has gone largely unstudied. These decisions occur in the context of economic risk, or variability in the value expected to accrue from the decision, and signal-borne risk, or ambiguity in the cues used to distinguish one option from another. Using an integrated framework like the ISE Framework, researchers can examine the influence of economic risk in signal detection issues where it has been underemphasized, for example in memory or perceptual learning, where costs and benefits are seldom modeled. Using an integrated framework, researchers could also examine the influence of signal-borne risk in economic issues where perceptual uncertainty has been underemphasized. For example, how do decision makers discriminate among what appear to be similar investment options, and how can emphasizing particular investment details (the equivalent of perceptual features) impact choices among investments with similar outcome variability? Furthermore, contextual factors, including individual psychological and biological differences and environmental influences, affect how perceivers make decisions about otherwise identical options. The integration of these decision features within a single framework can enhance our ability to understand a wide variety of decisions, in particular those complexly determined, real-world decisions that we make every day.

## Author contributions

SL and JW were responsible for the ISE Framework model. SL, JW, and KQ wrote the manuscript. LB contributed intellectual content and critically reviewed the manuscript.

### Conflict of interest statement

The authors declare that the research was conducted in the absence of any commercial or financial relationships that could be construed as a potential conflict of interest.

## Footnotes

^1^On the surface, choosing one of two (or more) options presented to a decision maker simultaneously might seem like a very different process than categorizing each of a series of individual signals presented one at a time. Nonetheless, categorizing a signal *is* a choice between one of two options: a perceiver decides to categorize the signal as Option A or Option B, and this decision is followed by an outcome. Moreover, SDT has a well-developed mathematical treatment of “simultaneous” presentation of two options, known as two-alternative forced-choice (2AFC; or more generally, *n*AFC, Macmillan and Creelman, 2005). Although we will continue to frame our discussion of SDT in terms of identification paradigms where stimuli are presented one at a time, the integration of the two frameworks readily translates to other SDT paradigms, including detection and *n*AFC.

^2^Perceivers do encounter variability in the outcome value they experience from responding to a particular signal value: when a signal (e.g., 40% scowling, in Figure 4) is an example of a target, choosing the “target” option will accrue a correct detection payoff (e.g., “That was correct.” feedback); when the signal is instead a foil, choosing the “target” option will accrue a false alarm payoff (e.g., “That was not correct.” feedback). This source of outcome value variability might be termed “selection risk” and is the recognized source of response bias in the signals framework. However, in contrast to the economic framework, selection risk is not intrinsic to the option itself (i.e., the targets do not vary in the benefit accrued to the perceiver for correctly responding “target”). Targets and foils do have two values associated with them (i.e., for targets, a benefit of correct detection and a cost of missed detection). However, in contrast to the economic framework, those values accrue only as a result of two different decisions (choose “target” vs. choose “foil,” respectively). They do not represent two different outcome values for the same choice, i.e., outcome values that are intrinsic to the target or foil category. Economic risk that is intrinsic to the target or foil category might be termed “category risk”: variability in a particular payoff (i.e., *h*, *j*, *a* or *m*; see Equation 4), inherent to the target or foil categories. Category risk is not typically manipulated within the signals framework. In the economic framework, selection risk and category risk are inseparable due to the lack of perceptual uncertainty (see Adding Perceptual Uncertainty to the Economic Framework).

^3^A component of our example that is relatively novel to the signals framework is that the signals here (what the weather looks like) are not emitted by the options themselves (biking and driving). Outside the laboratory, the perceptual domain on which decisions may be based can be separate from the options under consideration. Similarly, the perceptual cue can influence costs and benefits of options (i.e., getting rained on is a cost, directly associated with the perceptual cue of the current weather), whereas other costs and benefits are not related to the cue (e.g., benefit of exercise, cost of gas). Rather, they are inherent to the options, regardless of signal value.
